# PLK1 inhibitors for the treatment of colorectal cancer

**DOI:** 10.1097/MS9.0000000000003373

**Published:** 2025-05-20

**Authors:** Peng Ye, Zhen Zhang, Dingyue Zhang, Mingyue Zhang, Mengping Wang, Peiling Cai, Ying Huang, Yongyan Song

**Affiliations:** a Clinical Medical College & Affiliated Hospital & College of Preclinical Medicine, Chengdu University, Chengdu, China; b College of Preclinical Medicine, Chengdu University, Chengdu, China; c Xindu Hospital of Traditional Chinese Medicine, Chengdu, China

**Keywords:** anti-tumor therapy, colorectal cancer, PLK1 inhibitor

## Abstract

Polo-like kinase 1 (PLK1) is a key regulator in mitosis and a highly potent target for anti-cancer therapies. Several PLK1 inhibitors have been developed and evaluated for cancer treatment. However, none of them has yet been approved for clinical usage, mostly due to their low response rates in cancer patients. A recent phase I trial reported a 44% partial response rate of onvansertib, a PLK1 inhibitor, in the treatment of patients with *KRAS*-mutated metastatic colorectal cancer, indicating that PLK1 inhibitor might be suitable for the treatment of this specific subtype of cancer. This review summarizes the results of preclinical experiments and clinical trials of PLK1 inhibitors, with colorectal cancer as a focus, in hope of facilitating future investigations in this research field.

## Introduction

**Figure 1.**
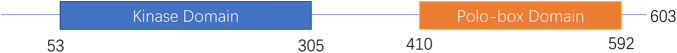
Schematic representation of PLK1.

Polo-like kinases (PLKs) are a family of serine/threonine kinases in humans which are homologous to polo, a serine/threonine kinase found in *Drosophila*^[1]^. Among the five members of PLKs family, polo-like kinase 1 (PLK1) was the first discovered and also the most intensively studied member. Several previous reviews have well summarized the structure and functions of PLK1^[2–8]^. Briefly, PLK1 protein contains 603 amino acids, with a characterized Polo-box domain at the C-terminal and a highly-conserved kinase domain at the N-terminal^[5]^ (Fig. 1). PLK1 plays key regulatory roles in several stages of cell mitosis (e.g. cytokinesis, centrosome maturation, chromosome segregation, spindle assembly, and sister chromatid separation), as well as in DNA damage checkpoint^[2,7,8]^. In addition, PLK1 controls the G2/M stop in cell cycle, and could trigger the onset of mitotic entry and therefore promote cell proliferation^[8]^. Overexpression of PLK1 was observed in many types of cancer including colorectal cancer (CRC)^[9]^, and was often associated with poor prognosis of patients^[10]^, indicating a role of PLK1 in tumorigenesis^[2,11]^. Due to its key roles in mitosis and DNA damage checkpoint and its overexpression in cancer, PLK1 is considered as a potential therapeutic target for cancer treatment^[2–5,7,11]^. Until now, although no one has yet been approved for clinical usage, several PLK1 inhibitors have been developed for the treatment of cancer, for example, BI2536^[12]^, Volasertib (BI6727)^[13]^, GSK461364^[14]^, HMN-214^[15]^, TKM-080301^[16]^, Rigosertib (ON01910)^[17]^, Scytonemin^[18]^, SBE-13^[19]^, LFM-A13^[20,21]^, Poloxin^[22]^, Purpurogallin^[23]^, TAK-960^[24]^, GW843682X^[25]^, ZK-thiazolidinone^[26]^, NMS-1^[26]^, CYC-800^[26]^, DAP-81^[26]^, and Onvansertib (PCM-075, NMS-1286937, NMS-P937)^[27]^ (see Table 1 for details).

**HIGHLIGHTS**

- Several Polo-like kinase 1 (PLK1) inhibitors have been developed and evaluated for cancer treatment.
- BI2536, volasertib, and GSK461364 showed limited clinical efficacy in treating colorectal cancer (CRC).
- HMN-214, poloxin, TAK-960, and rigosertib also showed limited clinical efficacy.
- Partial response was only observed in onvansertib in treating *KRAS*-mutated metastatic CRC (mCRC).
- *KRAS*-mutated mCRC might be a breakthrough in the development of PLK1 inhibitors.

**Table 1:** List of PLK1 inhibitors

PLK1 inhibitor	Developer	Mechanism of action
BI2536	Boehringer Ingelheim	block the ATP-binding site of PLK1 kinase domain
Volasertib (BI6727)	Boehringer Ingelheim	block the ATP-binding site of PLK1 kinase domain
GSK461364	GlaxoSmithKline	block the ATP-binding site of PLK1 kinase domain
HMN-214	Nippon Shinyaku	interfere with the spatial distribution of PLK1 inside the cell
Rigosertib (ON01910.Na)	Traws Pharma (formerly Onconova Therapeutics)	compete for the substrate-binding site of PLK1
Poloxin	/	inhibit the function of the PBD of PLK1
TAK-960	Takeda (Millennium) Pharmaceuticals	block the ATP-binding site of PLK1 kinase domain
Onvansertib (PCM-075, NMS-1286937, NMS-P937)	Cardiff Oncology (formerly called TrovaGene)	block the ATP-binding site of PLK1 kinase domain
TKM-080301	Tekmira Pharmaceuticals	siRNA against PLK1
Scytonemin	/	inhibit the activation of downstream signaling factors of PLK1
SBE-13	/	potent inhibitor of PLK1 in its inactive conformation
LFM-A13	/	bind to the catalytic site of the PLK1 kinase domain
Purpurogallin	/	PKD inhibitor of PLK1
GW843682X	/	ATP-competitive inhibitor of PLK1 and PLK3
ZK-thiazolidinone	Bayer	ATP-competitive inhibitor of PLK1
NMS-1	Nerviano Medical Science	selective PLK1 inhibitor
CYC-800	Cyclacel	selective PLK1 inhibitor
DAP-81	Rockefeller University	target PLKs, destabilizing kinetochore microtubules

CRC is the third most commonly diagnosed cancer and the second most common cause of cancer-related death worldwide, leading to over 0.9 million deaths in 2022^[28,29]^. Surgery remains the mainstay of treatment for CRC, and for non-resectable tumor, chemotherapy and radiotherapy are most commonly used^[30–32]^. Other treatment options for CRC include targeted therapies against vascular endothelial growth factor (e.g. bevacizumab), and targeted therapies against epidermal growth factor receptor (EGFR) (e.g. cetuximab and panitumumab) which are only suitable for *KRAS*-wildtype metastatic CRC (mCRC)^[33]^. In a recent phase Ib/II clinical trial, onvansertib, a PLK1 inhibitor, was used to treat *KRAS*-mutated mCRC in combination with chemotherapy and bevacizumab. The released results showed a partial response rate of 44% (8 out of 18) and median response duration of 9.5 months, and further phase II investigation is currently ongoing^[27]^.

Here, in this review, we summarize the preclinical and clinical results of each PLK1 inhibitor, with a focus on CRC, in hope of facilitating the future development of PLK1 inhibitors for the treatment of this disease. We first searched “PLK1 inhibitor” in Pubmed and “PLK1” in ClinicalTrials.gov, and from the results, we generated a list of PLK1 inhibitors. Names of each PLK1 inhibitor from the list were then searched in Pubmed, together with the keyword “colorectal cancer.” From the resulting articles organized by Endnote, the preclinical and clinical results of each PLK1 inhibitor targeting CRC were summarized below.

## BI2536

BI2536 is one of the earliest PLK1 inhibitor developed by Boehringer Ingelheim in 2007^[12]^. It is a small-molecule inhibitor, and works by blocking the ATP-binding site at the kinase domain of PLK1. BI2536 is a highly specific inhibitor and could inhibit PLK1 enzyme activity at nanomolar concentrations^[12]^. This compound was tested in different types of human cancer cell lines, including three colon cancer cell lines (HCT116 which was *KRAS*-mutated, and COLO205 and HT-29 which were *KRAS*-wildtype). The results showed that BI2536 could block the proliferation of these three types of human colon cancer cell lines, and the EC_50_ were all below 10 nM^[12]^. Further *in vivo* experiment showed that BI2536 given by intravenous injection could block the growth of human cancer xenografts in immunodeficient nude mice. In one of the colon cancer cell line, HCT-116, use of BI2536 twice a week resulted in complete suppression of tumor xenografts^[12]^.

In a phase I dose escalation and pharmacokinetic study, the maximum tolerated dose (MTD) of BI2536 was defined as 200 mg, and 23% of 40 patients enrolled showed disease stabilization for at least 3 months at MTD or higher doses^[34]^. There were in all 17 patients with CRC enrolled in this phase I study. In these 17 patients with CRC, 2 (11.8%) patients showed stable disease for 3 months or longer after BI2536 treatment^[34]^.

In another phase I study, BI2536 was given in two different dosing schedules (days 1 and 8 dosing schedule or day 1/24-h dosing schedule, with 21 days as a treatment course)^[35]^. The MTD of the first dosing schedule was defined as 100 mg per administration (200 mg per course), and MTD of the second dosing schedule was not determined. In days 1 and 8 dosing schedule group, 32% (14 out of 44) of the patients showed best overall response of stable disease, with no complete or partial response observed, and five patients showed no disease progression 3 months after BI2536 treatment^[35]^. In all, there were nine patients with CRC enrolled in the days 1 and 8 dosing schedule group. However, all of these patients showed disease progression 3 months after the treatment^[35]^.

Another phase I study used a different dosing schedule for BI2536, which was given in days 1–3 of a three-week treatment course^[36]^. MTD of this dosing schedule was defined as 60 mg. In the 21 patients enrolled (including 6 patients with CRC), no complete or partial response was observed, and 8 (38%) patients showed stable disease, and 6 of these patients remained progression-free 3 months after the treatment^[36]^. However, no specific efficacy results were disclosed for CRC^[36]^.

Possibly due to a lack of efficacy in patients with CRC in phase I clinical trials, further phase II trials of BI2536 were focusing on other types of cancer^[26]^.

## Volasertib (BI6727)

Similar to BI2536, volasertib (or named BI6727) was also a small-molecule PLK1 inhibitor (dihydropteridinone) developed by Boehringer Ingelheim. Volasertib also targets the ATP-binding site at the kinase domain of PLK1^[13]^. In the *in vitro* experiments, volasertib inhibited the growth of many types of cancer cell lines, including colon cancer cell line (HCT116) with EC_50_ of 23 nM^[13]^. In *in vivo* experiments, treatment of volasertib resulted in significantly delayed tumor growth in nude mice bearing HCT116 human colon cancer xenograft models. In addition, volasertib also significantly delayed the growth of CXB1 human colon cancer xenograft models which were taxane-resistant (e.g. resistant to docetaxel)^[13]^.

In a phase I, dose-escalation study, 65 patients received volasertib treatment, including 8 (12%) patients with CRC. Results showed that three (5%) patients had partial response, but none of them had CRC^[37]^. In the 65 patients, 26 (40%) had stable disease as the best overall response. Detailed cancer types of these 26 patients were, however, not disclosed^[37]^. The MTD of volasertib was defined as 400 mg in this phase I clinical trial.

In another phase I trial, 59 patients with advanced cancer received treatment of volasertib, including 11 patients with CRC^[38]^. Two patients with ureteral urothelial carcinoma or melanoma showed partial response, and another 26 (44%) patients showed stable disease. Similarly, no detailed cancer type of these 26 patients was disclosed^[38]^.

In a third phase I trial for volasertib, 61 patients received treatment of volasertib in combination with cisplatin or carboplatin^[39]^. The MTD of volasertib was determined to be 300 mg. In the 30 patients treated with volasertib and cisplatin, two (6.7%) patients with dendritic reticulum cell sarcoma showed partial response, and another 11 (36.7%) patients showed stable disease. There were four patients with CRC in this treatment arm, and three of them showed stable disease. In the other treatment arm, 31 patients were treated with volasertib and carboplatin, including 4 patients with CRC. Two (6.5%) patients showed partial response, including one patient with poorly differentiated hypopharynx carcinoma and the other patient with squamous non-small cell lung cancer. Another six patients showed stable disease, but the detailed cancer types for these six patients were not disclosed^[39]^.

In a fourth phase I trial, 57 patients were treated with a combination of volasertib and afatinib, an EGFR inhibitor^[40]^. The MTD determined was 300 mg volasertib plus 30 mg afatinib (on days 2–21) or 70 afatinib (on days 2–6). In the 29 patients treated with volasertib plus afatinib on days 2–21, 2 (6.9%) patients with non-small cell lung cancer or head and neck cancer achieved partial response, and 8 (27.6%) patients achieved stable disease (detailed cancer types not disclosed). Seven patients with CRC were enrolled in the treatment arm. In the other treatment arm, 28 patients were treated with volasertib plus afatinib on days 2–6, including 16 patients with CRC. No complete or partial response was observed, and 8 (28.6%) patients showed stable disease (detailed cancer types not disclosed)^[40]^.

In a fifth phase I trial of volasertib, 30 patients were treated with volasertib in combination with nintedanib^[41]^. The MTD was 300 mg for volasertib, with 3 weeks as a treatment course. Complete response was observed in a patient with breast cancer, and partial response was observed in a patient with non-small cell lung cancer. Stable disease was observed in 16 patients. Ten patients with CRC were enrolled in this trial, and five of them showed stable disease after treatment^[41]^.

In a sixth-phase I trial of volasertib, 28 patients (including 7 patients with CRC) were treated with volasertib plus itraconazole, a P-glycoprotein and cytochrome P-450 3A4 inhibitor^[42]^. Twenty-five (89%) patients achieved stable disease, with no complete or partial response observed. Detailed cancer types of these 25 patients were not disclosed.

In another two-phase I trials, volasertib was evaluated in patients with solid tumors, but no patients with CRC were recruited^[43,44]^.

Phase II trials for volasertib have been conducted in patients with metastatic urothelial cancer^[45]^ or acute leukemia^[46]^, and a phase III trial was conducted in patients with acute leukemia^[47]^. No phase II or III trials have been conducted for patients with CRC.

## GSK461364

GSK461364 is a small-molecule inhibitor for PLK1 developed by GlaxoSmithKline. It also works by binding to the ATP-binding site at the kinase domain of PLK1 and inhibiting the enzyme activity of PLK1^[14]^. The preclinical results of GSK461364 were not published, and a phase I clinical trial has evaluated the safety and efficacy of GSK461364 in advanced solid tumors^[14]^. In all, there were 40 patients enrolled, including 12 patients with CRC, who were treated with GSK461364 on days 1, 8, and 15 of 28-day cycles (schedule A), or on days 1, 2, 8, 9, 15, and 16 of 28-day cycles (schedule B). The MTD was determined to be 225 mg for schedule A, and 75 mg for schedule B. Six patients achieved stable disease as best tumor response, and none of these patients had CRC. No complete or partial response was observed. Further clinical investigations were not conducted.

## HMN-214

HMN-214 is a PLK1 inhibitor developed by Nippon Shinyaku. It is a stilbene derivative which inhibits PLK1 by interfering with the spatial distribution of PLK1 inside the cell^[15]^. In a phase I dose-escalation study, 33 patients were treated with HMN-214, including 13 patients with CRC. In the 29 patients who finished response assessment, 7 patients achieved stable disease, with no complete or partial response observed^[48]^. Further investigation was discontinued.

## Rigosertib (ON01910.Na)

Rigosertib (ON01910.Na) is a small-molecule inhibitor for PLK1 developed by Traws Pharma (former Onconova Therapeutics and Trawsfynydd Therapeutics). It is believed that rigosertib functions by competing for the substrate-binding site of PLK1, and inhibits the activation of downstream signaling factors of PLK1, for example, CDC25C^[17]^. In addition, rigosertib was also found to inhibit phosphoinositide 3-kinase (PI3K) pathway^[49]^. *In vitro* experiments showed that rigosertib could inhibit growth of cancer cell lines, including four CRC cell lines (COLO-205, DLD-1, HCT15, and SW480) with IC_50_ of 125–250 nM^[50]^. In a recent study, rigosertib showed stronger therapeutic effects in *KRAS*-mutant, chemotherapy-resistant patient-derived CRC xenograft, compared to the standard therapy (fluoropyrimidine plus oxaliplatin/irinotecan plus bevacizumab), indicating potential of PLK1 inhibitors in the treatment of *KRAS*-mutated CRC^[51]^. In another study, rigosertib was shown to promote the anti-tumor immunity through degradation of PD-L1 in CRC cell lines^[52]^.

In a phase I trial, 20 patients with solid tumors received rigosertib treatment on days 1, 4, 8, 11, 15, and 18 of a 28-day treatment course, including 7 patients with CRC. The MTD was defined as 3120 mg for rigosertib. In the 16 patients with assessable response, 1 patient with hepatocellular cancer achieved stable disease, and 1 patient with ovarian cancer achieved partial response, and the rest patients had the best response of progressive disease^[17]^.

In another phase I trial, 46 patients with solid tumors were treated with combination of rigosertib and gemcitabine, including 1 patient with CRC^[49]^. The rigosertib was also given on days 1, 4, 8, 11, 15, and 18 of a 28-day treatment course. Three patients with Hodgkin lymphoma, thymic cancer, or pancreatic ductal adenocarcinoma achieved partial response^[49]^.

In a third phase I trial, 29 patients were treated with rigosertib for 3 continuous days, with 2 weeks as a treatment course^[53]^. In the 22 patients with evaluable response, 9 (41%) patients showed stable disease as their best response, and no complete or partial response was observed. Five patients with CRC were enrolled in this trial, and none of them achieved stable disease^[53]^.

In a fourth phase I trial, 48 patients were given rigosertib twice daily continuously in 21-day cycles, including 10 patients with CRC^[54]^. The recommended phase II dose was determined to be 560 mg twice daily. Two patients with head and neck squamous cell carcinoma achieved complete response or partial response. Another eight patients achieved stable disease, but detailed cancer types of these patients were not disclosed^[54]^.

In a fifth phase I trial of rigosertib, 25 patients with advanced cancer were given rigosertib treatment by 2-, 4-, or 8-hour infusion twice a week for the first 3 weeks of a 4-week treatment course. Only one patient with CRC was enrolled in the trial. In the 14 patients with evaluable response, the best response observed was stable disease in 5 (35.7%) patients with thymoma, hepatocellular carcinoma, pancreatic cancer, gallbladder cancer, and chronic myelocytic leukemia^[55]^.

Further phase II/III investigation for rigosertib was conducted in patients with previously untreated metastatic pancreatic cancer^[56]^, but not CRC.

## Poloxin

Poloxin is a small-molecule inhibitor that specifically targets the function of the PBD of PLK1^[22]^. Poloxin could induce fragmentation of centrosome, abnormal spindle, and chromosome misalignment, which cause mitotic arrest and cell apoptosis. *In vitro* experiments showed that poloxin could inhibit the growth of cancer cell lines, including two CRC cell lines, SW480 and HCT116, with EC_50_ of 22 and 25 µM, respectively^[57]^. Unfortunately, no further clinical investigation has been conducted for this compound.

## TAK-960

Developed by Takeda (Millennium) Pharmaceuticals, TAK-960 is a small-molecule inhibitor which could competitively bind to the ATP-binding domain of PLK1^[24]^. TAK-960 was found to inhibit the proliferation of human cancer cell lines regardless of *TP53* and *KRAS* mutation status, including several CRC cell lines, for example, HT-29, HCT116, COLO320DM, HCT-15, RKO, SW480, and SW620. *In vivo* experiments showed that TAK-960 also significantly delayed the growth of tumor xenografts, including those derived from CRC cell lines (HCT116, HT-29)^[58]^. Further *in vivo* experiments using patient-derived xenograft models showed that TAK-960 treatment was effective in 6 out of 18 CRC xenograft models, which was not associated with the *KRAS, BRAF, NRAS, PIK3CA*, or *TP53* mutation status^[59]^.

TAK-960 was evaluated in a phase I trial in patients with advanced non-hematologic malignancies (NCT01179399). The compound was given orally for 21 days of a 28-day treatment course. Unfortunately, the trial was discontinued due to pipeline prioritization^[60]^, and no efficacy data were released.

## Onvansertib (PCM-075, NMS-1286937, NMS-P937)

Onvansertib (PCM-075, NMS-1286937, NMS-P937) is a small-molecule, highly-selective PLK1 inhibitor developed by Cardiff Oncology (formerly called TrovaGene). It is a pyrazoloquinazoline which could compete with ATP and inhibit the kinase activity of PLK1, with IC50 of 2 nM^[61,62]^. Onvansertib could suppress the growth of human cancer cells *in vitro*, including CRC cell lines. It also showed good anti-tumor activity against human colon cancer (HCT116)-derived xenografts in mice^[63]^. In a phase I, dose escalation study which was conducted between December 2009 and April 19, 2012, patients with advanced/metastatic solid tumors (including four patients with CRC) were given treatment of onvansertib orally for 5 consecutive days in a 21-day treatment cycle^[64]^. Out of the 16 patients with evaluable efficacy, 5 (31.2%) patients achieved stable disease, including 2 patients with CRC, 1 patient with *KRAS*-mutated pancreatic carcinoma, 1 patient with head and neck squamous cell carcinoma, and 1 patient with basal cell carcinoma^[64]^. Further phase II investigation was not conducted.

Later, preclinical studies found that PLK1 inhibition could enhance the efficacy of oxiplatin and irinotecan in preclinical models of CRC^[27,65,66]^. In addition, a genome-wide RNAi screen showed that PLK1 is synthetically lethal with mutated *KRAS* in CRC cells^[67]^. Further preclinical experiments showed that onvansertib was more effective in *KRAS*-mutated CRC cells^[27]^. Compared to single agents of onvansertib and irinotecan, combination of the two treatments also showed stronger suppression on the growth of *KRAS*-mutated HCT116 CRC xenograft model^[27]^. On the basis of these findings, investigators conducted a phase Ib/II trial to evaluate the safety and efficacy of a combination of onvansertib and standard of care (folinic acid, fluorouracil, and irinotecan, plus bevacizumab) in *KRAS*-mutated mCRC. As mentioned previously, the released results of the phase Ib trial showed that in the 18 patients enrolled, 8 (44%) patients achieved partial response, with median response duration of 9.5 months. Further phase II trial is currently ongoing (NCT03829410).

## Others

In some of the PLK1 inhibitors, preclinical experiments were not conducted in CRC cell lines or xenografts, for example, TKM-080301, Scytonemin, SBE-13, LFM-A13, Purpurogallin, GW843682X, ZK-thiazolidinone, NMS-1, CYC-800, DAP-81, and LC-445. Details of these inhibitors were therefore not discussed in this review.

## Conclusions

In this review, we summarized preclinical and clinical study results of eight most well-investigated PLK1 inhibitors which were evaluated in patients with CRC. As illustrated in Table 2, most of the clinical trials were performed in Caucasian population, and the inclusion criteria were similar between different trials, except for onvansertib which required KRAS mutation in exons 2, 3, or 4^[27]^. BI2536, volasertib, and rigosertib were intensively investigated in phase I trials, either as single regimen or in combination with other therapies. However, probably due to lack of efficacy in phase I trials of these three PLK1 inhibitors, no further phase II trials were conducted in patients with CRC. Possible reasons for the lack of efficacy in these PLK1 inhibitors could be: (1) some PLK1 inhibitors have multiple targets (e.g. BI2536 also targets BRD4^[68]^, and Rigosertib also inhibits PI3K^[49]^) which may increase the chance of side effects, and these side effects may become intolerable before any efficacy was shown; (2) the patient selection performed in these phase I clinical trials may not be enough to identify potential patient subgroups who benefit from the treatment. In all the clinical trial results of the eight PLK1 inhibitors discussed in this review, partial response was only observed in onvansertib for the treatment of patients with *KRAS*-mutated mCRC, where patient selection was used. Future investigations should focus on the development of highly specific PLK1 inhibitors, and the genetic background of tumors of the patients should be intensively analyzed to maximize the chance of finding potential patient subgroups benefiting from the treatment.

**Table 2:** Phase I trial results of patients with CRC evaluating PLK1 inhibitors

PLK1 inhibitor	N of patients with CRC	Race of patient population	Inclusion criteria	Treatment	Efficacy results
BI2536^[34]^	17	Caucasian	adult; refractory advanced and/or metastatic solid tumors; life expectancy ≥6 months; Eastern Cooperative Oncology Group (ECOG) ≤2	single-dose	stable disease in 2 (11.8%) patients for 3 months or longer
BI2536^[35]^	9	Caucasian	adult; confirmed diagnosis of advanced, non-resectable, and/or metastatic solid tumors and evaluable tumor deposits; failed conventional treatment; life expectancy ≥6 months; ECOG ≤ 2	days 1 and 8 dosing schedule	disease progression in all the 9 patients 3 months after treatment
BI2536^[36]^	6	Caucasian	adult; confirmed diagnosis of advanced non-resectable or metastatic solid tumours (or both) and evaluable tumour deposits; ECOG ≤2	days 1–3 of a 3-week cycle	details not disclosed (stable disease or disease progression)
volasertib^[37]^	8	Caucasian	adult; confirmed diagnosis of advanced, non-resectable and/or metastatic solid tumours; failed established treatments; ECOG ≤2	single infusion every 3 weeks	details not disclosed (stable disease or disease progression)
volasertib^[38]^	11	Asian	adult; histologically or cytologically confirmed diagnosis of advanced, non-resectable, and/or metastatic solid cancer, who had failed conventional treatment, or for whom no therapy of proven efficacy existed, or who were not amenable to established forms of treatment; ECOG ≤2; recovery from toxicities from previous treatments; adequate bone marrow, liver, and renal function; life expectancy ≥6 months	single infusion every 3 weeks	details not disclosed (stable disease or disease progression)
volasertib^[39]^	4	Caucasian	adult; confirmed diagnosis of advanced, non-resectable or metastatic solid tumors, who had failed conventional treatment, or for whom no therapy of proven efficacy existed, or who were not amenable to established forms of treatment; indication for treatment with platinum therapy as judged by the investigator; ECOG ≤2; recovery from Common Terminology Criteria for Adverse Events (CTCAE) grades 2–4 therapy-related Adverse Events from previous systemic anticancer therapies or radiotherapies (except alopecia of CTCAE grade 2)	volasertib + cisplatin	stable disease in 3 patients
volasertib^[39]^	4			volasertib + carboplatin	details not disclosed (stable disease or disease progression)
volasertib^[40]^	7	Caucasian	adult; with a histologically or cytologically confirmed diagnosis of an advanced, non-resectable and/or metastatic relapsed or refractory solid tumor after failure of conventional treatment, or patients who were not amenable to standard therapy, or for whom no therapy of proven efficacy existed; ECOG ≤2; have recovered from clinically significant toxicities from previous systemic anticancer therapies or radiotherapies (except alopecia grade 2)	volasertib + afatinib on days 2–21	details not disclosed (stable disease or disease progression)
volasertib^[40]^	16			volasertib + afatinib on days 2–6	details not disclosed (stable disease or disease progression)
volasertib^[41]^	10	Caucasian	adult; confirmed diagnosis of advanced, metastatic solid tumors following failure of conventional treatment, for whom no therapy of proven efficacy existed or who were not amenable to established forms of treatment; ECOG ≤2; recovery from CTCAE grades 2–4 therapy-related toxicities from previous systemic anticancer therapies or radiotherapy (except alopecia)	volasertib + nintedanib	stable disease in five patients
volasertib^[42]^	7	Caucasian	aged 18–70 years; ECOG ≤2; confirmed diagnosis of advanced, non-resectable, and/or metastatic solid tumor; either failed conventional treatment, had no therapy of proven efficacy available, or were not amenable to established forms of treatment, based on the investigator’s assessment	volasertib + itraconazole	details not disclosed (stable disease or disease progression)
GSK461364^[14]^	12	Caucasian	adult; confirmed diagnosis of advanced solid tumor for which no effective treatment was available; ECOG ≤2; adequate bone marrow, renal, and hepatic functions; CTCAE grade ≤1; prior anticancer treatment ≥3 weeks of study entry; absence of significant comorbidities	once or twice a week of 28-day cycles	disease progression in all the patients with CRC
HMN-214^[48]^	13	Caucasian	adult; pathologically confirmed advanced solid malignancy for which no standard treatment existed or that had progressed or recurred following prior therapy; life expectancy >12 weeks; ECOG ≤2; adequate hematopoietic, hepatic, and renal functions	oral administration on days 1 and 21 of a 28-day cycle	details not disclosed (stable disease or disease progression)
rigosertib^[17]^	7	Caucasian	adult; histologically confirmed malignancy for which standard curative or palliative measures did not exist; ECOG ≤2; life expectancy ≥12 weeks; adequate bone marrow, hepatic, and renal function; any prior therapy ending 28 days before study without residual toxicity	rigosertib on days 1, 4, 8, 11, 15, and 18 of a 28-day cycle	disease progression in all the patients with CRC
rigosertib^[49]^	1	Caucasian	adult; histologically confirmed solid malignancy (including Hodgkin lymphoma) for which standard curative or palliative measures did not exist or were no longer effective or patients with a clinical rationale; life expectancy >12 weeks; ECOG ≤2; have evaluable disease, either with tumor markers or with measurable disease in imaging by Response Evaluation Criteria in Solid Tumors (RECIST); adequate bone marrow, hepatic, and renal function; completed radiotherapy and/or chemotherapy more than 4 weeks before study drug initiation and without residual toxicity	rigosertib + gemcitabine	disease progression in all the patients with CRC
rigosertib^[53]^	5	Caucasian	adult; histologically confirmed malignancy that was metastatic or unresectable and for which standard curative or palliative measures did not exist or were no longer effective; at least 4 weeks since the last dose of potentially myelosuppressive treatment; have recovered from reversible drug toxicities (except alopecia, stable residual neuropathy, and residual hand-foot syndrome); ECOG ≤2; nearly normal organ and bone marrow function	3 continuous days in 2-week cycle	disease progression in all the patients with CRC
rigosertib^[54]^	10	Caucasian	adult; incurable, histologically confirmed solid malignancy; ECOG ≤2; life expectancy ≥6 months; measurable disease per RECIST; adequate bone marrow, hepatic and renal function; Surgery or palliative radiotherapy ≥14 days or chemotherapy >21 days before treatment without residual grade >1 toxicity	twice daily continuously in 21-day cycles	details not disclosed (stable disease or disease progression)
rigosertib^[55]^	1	Asian	not disclosed	twice a week for the first 3 weeks of a 4-week cycle	disease progression in all the patients with CRC
onvansertib^[64]^	4	Caucasian	not disclosed	orally for 5 consecutive days in a 21-day cycle	stable disease in 2 patients with CRC
onvansertib^[27]^	18	Caucasian	adult; histologically confirmed metastatic and unresectable colorectal cancer and a KRAS mutation in exon 2, 3, or 4 in either the primary tumor or the metastasis; have received a minimum of 6 weeks of a first-line regimen that included oxaliplatin and a fluoropyrimidine, with or without bevacizumab, and to have failed treatment or have been intolerant to oxaliplatin; ECOG ≤2; adequate organ function; not having microsatellite instability high/deficient mismatch repair, BRAF V600 mutations, more than one prior chemotherapy regimen administered in the metastatic setting, or untreated brain metastasis	onvansertib + standard of care	partial response in 8 (44%) patients, median response duration of 9.5 months

## Data Availability

Data sharing is not applicable to this article.

## Research registration unique identifying number (UIN)

Not relevant to our manuscript.
